# Metabolic Crisis in Severely Head-Injured Patients: Is Ischemia Just the Tip of the Iceberg?

**DOI:** 10.3389/fneur.2013.00146

**Published:** 2013-10-11

**Authors:** Emilie Carre, Michael Ogier, Henry Boret, Ambroise Montcriol, Lionel Bourdon, Risso Jean-Jacques

**Affiliations:** ^1^Unit of Traumatology, Institut de Recherche Biomedicale des Armees, Bretigny, France; ^2^Intensive Care Unit, Hopital d’Instruction des Armees Sainte-Anne, Toulon, France

**Keywords:** metabolic crisis, ischemia, head injury, multimodal monitoring, intracerebral microdialysis

## Abstract

Ischemia and metabolic crisis are frequent post-traumatic secondary brain insults that negatively influence outcome. Clinicians commonly mix up these two types of insults, mainly because high lactate/pyruvate ratio (LPR) is the common marker for both ischemia and metabolic crisis. However, LPR elevations during ischemia and metabolic crisis reflect two different energetic imbalances: ischemia (Type 1 LPR elevations with low oxygenation) is characterized by a drastic deprivation of energetic substrates, whereas metabolic crisis (Type 2 LPR elevations with normal or high oxygenation) is associated with profound mitochondrial dysfunction but normal supply of energetic substrates. The discrimination between ischemia and metabolic crisis is crucial because conventional recommendations against ischemia may be detrimental for patients with metabolic crisis. Multimodal monitoring, including microdialysis and brain tissue oxygen monitoring, allows such discrimination, but these techniques are not easily accessible to all head-injured patients. Thus, a new “gold standard” and adapted medical education are required to optimize the management of patients with metabolic crisis.

Managing traumatic brain injury is like navigating the ocean. Dangers (secondary insults) are everywhere, but, usually, easy to prevent. In this ocean, just imagine cerebral energetic disturbances as an iceberg, where ischemia is the tip (Figure 1). All clinicians are aware of what ischemia is and how to prevent/manage ischemic events. However the real danger is the submerged part of the iceberg, the one we cannot even imagine. Metabolic crisis may represent that sneaky part of energetic post-traumatic disturbances: they are still not well understood, difficult to detect and to care.

**Figure 1:**
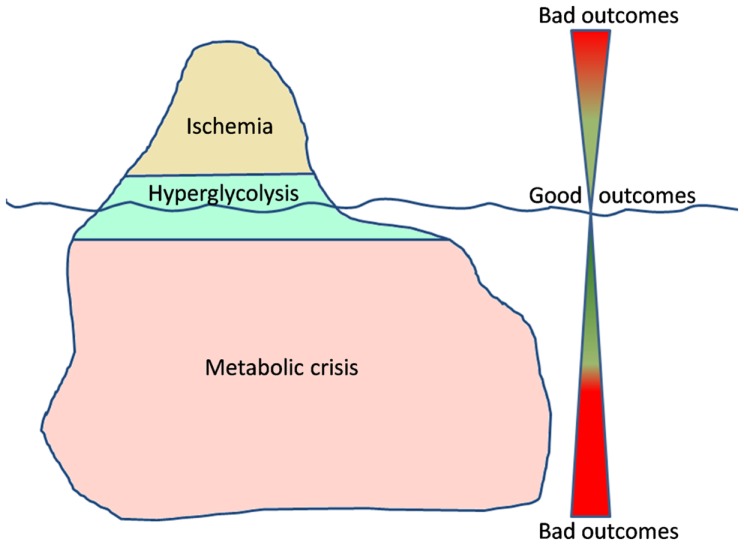
**The iceberg of metabolic disturbances associated with a high lactate/pyruvate ratio**.

## Confusing Definition of Metabolic Crisis in the Literature

In Intensive Care Unit (ICU), cerebral post-traumatic metabolic disturbances have commonly been characterized by an increase of the lactate/pyruvate ratio (LPR) above 40, as measured by clinical intracerebral microdialysis. High LPR has originally been attributed to compromised cerebral perfusion and impaired oxygen delivery. However, high LPR may actually reflect several pathological events or compensatory mechanisms (1). After traumatic brain injury, three different types of metabolic disturbances, all characterized by a LPR > 40, have been reported: hyperglycolysis, ischemia, and a pattern described by Vespa and colleagues initially called “metabolic crisis without brain ischemia” (2). Vespa’s initial study showed a 25% incidence of high LPR, but only a 2.4% incidence of ischemia (measured with positron emission tomography), in 19 brain injury patients. In this study, most of the episodes of high LPR were correlated with non-ischemic reduction in cerebral oxygen metabolism. This metabolic crisis may be the most frequent form of post-traumatic metabolic disturbance (2, 3). Indeed, 74% of head-injured patients may suffer from metabolic crisis in the first days after the initial trauma, despite successful resuscitation and tight control of their intracranial pressure (4). Prolonged state of metabolic crisis is associated with poor outcome at 6 months post-trauma as well as regional chronic brain atrophy (4–6). These metabolic crises have also been observed in patients with terminal herniation (7).

In the literature, confusion persists with articles dealing with metabolic crisis and that potentially refer to any type of metabolic disturbances (hyperglycolysis, ischemia, metabolic crisis …). The discrimination between different types of metabolic disturbances is, however, crucial because each type requires an adapted management to avoid deterioration of the patients. In addition, there is currently no consensual terminology for the specific pattern of metabolic disturbance characterized by elevated extracellular LPR and normal oxygenation. Indeed, the original work of Vespa and colleagues referred to this kind of disturbance as “metabolic crisis without brain ischemia” (2), whereas others called it “non-ischemic oxidative metabolic dysfunction” (6), “non-ischemic impairment of oxidative metabolism” (3), or “non-ischemic energy metabolic crisis” (8). Because different terminology could be misleading, we propose the unifying terminology: “metabolic crisis.”

## Metabolic Disturbances after Head Injury

To understand the singularities of each type of post-traumatic metabolic disturbance (hyperglycolysis, ischemia, and metabolic crisis), some physiological aspects of the cerebral metabolism need to be considered.

### Cerebral metabolism in physiological conditions

**Figure 2:**
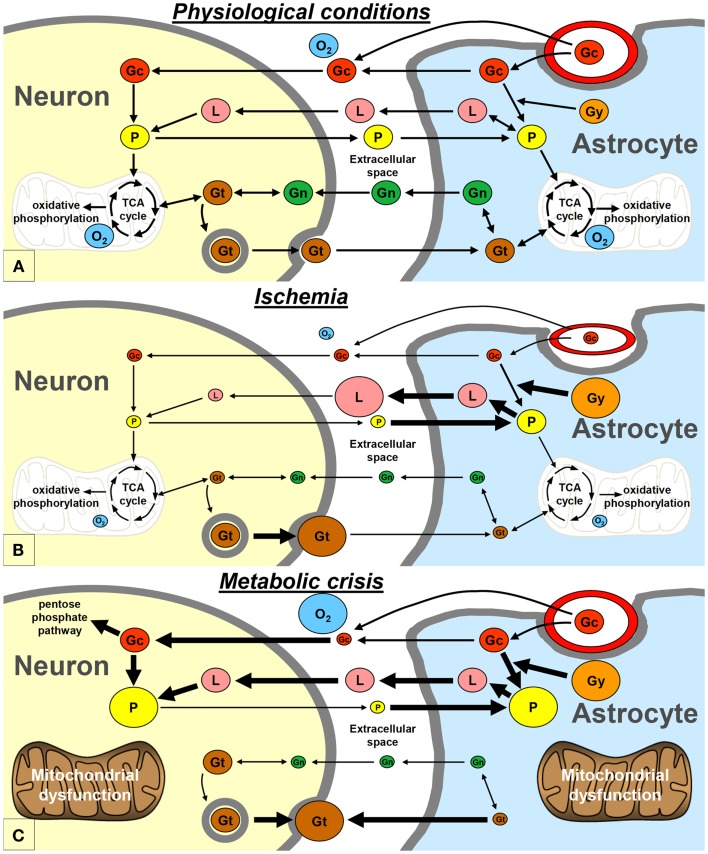
**Schematic representation of the proposed exchange of energetic metabolites between neurons and astrocytes, in physiological conditions (A), during ischemia (B), and during metabolic crisis (C)**. Gc, glucose; Gg, glycogen; Gn, glutamine; Gt, glutamate; L, lactate; O_2_, oxygen; P, pyruvate; TCA, tricarboxylic acid.

Neurons and astrocytes can efficiently utilize lactate, pyruvate, glutamate, and glutamine as energetic substrates, in addition to glucose (9). Astrocytes play a pivotal role in providing these energetic substrates to neurons (Figure 2A). The astrocyte-neuron lactate shuttle model of Pellerin and Magistretti (10) suggests that neuronal activity is tightly coupled to glucose utilization/glycolysis. Glutamate is released from active synapses and uptaken by astrocytes. Glutamate uptake stimulates the activity of the Na/K-ATPase and subsequently the entry of glucose from the vasculature into astrocytes. A large portion of the glucose entering the astrocytes is then directed to the glycolytic pathway and is metabolized into lactate which is released in the extracellular space. Lactate is then transported to neurons and converted to pyruvate that can be used as an energetic substrate through the tricarboxylic acid (TCA) cycle (10). In addition, part of the neuronal pyruvate is transferred to astrocytes in order to close the redox loop (11). Under certain circumstances, astrocytic glycogen stores may also be metabolized into lactate, which is subsequently released in the extracellular space (12). Astrocytes also play an important role in providing glutamate back to neurons through the glutamate-glutamine cycle. Indeed, glutamate is converted into glutamine in astrocytes; then, glutamine is transported to neurons where it is finally converted into glutamate (10). Glutamate and glutamine can also be metabolized through diverse pathways in astrocytes and neurons, especially through the TCA cycle (9).

### Compensatory hyperglycolysis

An increase in glucose utilization is commonly observed in head-injured patients in the first days after injury. Several roles have been proposed for this post-traumatic hyperglycolysis, including restoration of the ionic balance. This compensatory hyperglycolysis can induce a transient accumulation of lactate in the extracellular compartment of the brain in head-injured patients (2, 7). Under these conditions, lactate is likely to be used by neurons as an additional source of energy. This may explain why cerebral accumulation of extracellular “good” lactate, in absence of hypoxia, has been associated with good long-term recovery in subarachnoid hemorrhage patients (13). Other mechanisms may participate, in addition to hyperglycolysis, to restore the ionic homeostasis, such as an increase in the glutamate-glutamine cycle turnover (14–16).

### Metabolic disturbances during ischemia

It is commonly admitted that ischemia is one of the most deteriorating post-traumatic insults. Experimental studies of traumatic brain injury have shown that cerebral oxidative metabolism is reduced due to ischemia, because of oxygen and glucose deprivation (14, 16). In neurons, as a result of the lack of oxygen, pyruvate no longer enters the TCA cycle and the need for lactate may decrease (Figure 2B). On the other hand, pyruvate and glycogen are heavily metabolized into lactate in astrocytes (12). Therefore, lactate accumulates and pyruvate level decreases in the extracellular space, leading to an increase of the LPR. This pattern is known as “Type I” LPR elevation (8, 17) and has been associated with a poor outcome in head-injured patients (18). Type I LPR elevation can also be associated with high extracellular glutamate, as a consequence of the reversal of neuronal glutamate transporters (19).

### Metabolic crisis

Vespa’s article published in 2005 may be considered as the princeps article introducing the concept of the metabolic crisis (2). Metabolic crisis is characterized by a “Type 2” LPR elevation, which is due to a reduction in extracellular pyruvate level, with normal or elevated tissue oxygen level (Figure 2C) (8, 17). Occurrences of high extracellular glutamate and low glucose have also been reported during metabolic crisis (4, 7). This pattern may appear very similar to that of ischemia; however, the underlying mechanisms are profoundly different (see below).

## Suspected Mechanisms of Metabolic Crisis

There is a gap of knowledge regarding the exact mechanisms underlying metabolic crisis. Nevertheless, two main hypotheses have been proposed: mitochondrial dysfunction and excessive increase in metabolic demand.

As early as 1942, Lindquist proposed the hypothesis that “some more fundamental factor of disturbed physiology was responsible for the syndrome of head injury, and […] that the injured nerve cells might be unable to utilize oxygen normally in spite of an adequate oxygen supply” (20). More recently, clinical studies have demonstrated that some head-injured patient have disturbances in oxidative metabolism due to mitochondrial dysfunction, despite good oxygen supply (21, 22), suggesting that mitochondrial dysfunction may be one of the mechanisms underlying metabolic crisis. In experimental settings, however, mitochondrial dysfunction, induced by cyanide poisoning, is associated with an increase in LPR and brain tissue oxygen (P_ti_O_2_), but not with a decrease in extracellular pyruvate level (23, 24). Therefore, mitochondrial dysfunction *per se* may be a mechanism involved in metabolic crisis but it is not responsible for the decrease in extracellular pyruvate level. Instead, the low interstitial pyruvate measured during metabolic crisis may be the consequence of a shunting of the glycolytic pathway in favor of the pentose phosphate pathway, which plays a protective role in neutralizing oxygen free radicals. This shunting may limit oxidative mitochondrial damage (25). In addition, neuronal pyruvate may not be transferred to astrocytes anymore and might serve as a endogenous free radical scavenger to stabilize neuronal mitochondrial function (26).

Excessive increase in metabolic demand is the other proposed mechanism leading to metabolic crisis. Indeed, post-traumatic seizures and cortical spreading depression both result in excessive increases in metabolic demand (2, 27). Under these circumstances, neuronal and astrocytic hyperglycolysis may not be sufficient to compensate for the deficits in oxidative metabolism. In addition, when astrocytic glycogen stores are depleted, the resulting energetic failure can alter the activity of the Na/K-ATPase and lead to intracellular accumulation of Na+, consecutive astrocytic swelling and subsequent mitochondrial swelling, which may further alter mitochondrial function (28, 29). In response to swelling, astrocytic volume-regulated anion channels can open and allow the efflux of glutamate and other amino acids, as part of an osmoregulation process (30). A reversal of astrocytic glutamate transporters can also be observed (19). The resulting catastrophic surge of extracellular glutamate can lead to excitotoxic NMDA-receptor activation and mitochondrial calcium overload, which result in drastic mitochondrial depolarization and cellular death by apoptosis and/or necrosis (31). Furthermore, as a consequence of intracellular glutamate depletion in astrocytes, the glutamate-glutamine cycle turnover decreases (15), and glutamate and glutamine can no longer be used as energetic substrates.

It is difficult to determine which of mitochondrial dysfunction and excessive energetic demand, if any, is the initial trigger of metabolic crisis but we may assume that mitochondrial impairment enhances excessive energetic demand and that energy failure alters mitochondrial function. Here could be the vicious circle of metabolic crisis.

## Diagnosis and Treatment of Metabolic Crisis

The occurrence of metabolic crisis is associated with poor outcome after brain injury (4, 7, 32). It is therefore important to identify patients with metabolic crisis to optimize their management.

According to us, one of the main problems in the management of head-injured patients is the lack of knowledge about metabolic crisis. Indeed, medical education about metabolic crisis is still very limited. The last Brain Trauma Foundation guidelines, in particular, do not mention metabolic crisis or any similar metabolic pattern (33). Neurointensivists are usually familiar with multimodal monitoring and have consequently at least some knowledge of metabolic crisis. On the other hand, in “general” ICUs, intensivists, and medical staff are well-trained to treat patients with multi-organ failure, but some specificities in the management of neurocritical care patients may sometimes be under-recognized (34). Have all intensivists in “general” ICUs already heard of metabolic crisis? We are not so sure. As only 33% of the US population are within 90 min of a Neurocritical Care Unit (35), the vast majority of head-injured patients may be treated in “general” ICUs, where metabolic crisis is unlikely to be suspected, largely because of unawareness. We believe that an effort should be made in providing medical education about metabolic crisis in all of the ICUs.

Up to now, no therapeutic treatment has shown effectiveness in improving the outcome of patients with metabolic crisis. Indeed, in clinical studies, aggressive maintenance of cerebral perfusion pressure, or decrease of the intracranial pressure (with mannitol, hyperventilation, …), have unfortunately failed to improve the oxidative metabolism or normalized the biomarkers of metabolic crisis (4, 36–39). In addition, the treatments used to target ischemia may even be particularly deleterious in patients with metabolic crisis. Hyperoxia, in particular, does not improve cerebral oxygen utilization and may generate free radicals that can exacerbate mitochondrial dysfunction in these patients (40). According to Verweij, “Restoring mitochondrial function might be as important as maintaining oxygen delivery” in patients with severe brain injury (21). Permeabilization of the mitochondrial membrane may therefore represent a particularly interesting target for therapeutic strategies against metabolic crisis (29). Consistent with this hypothesis, recent studies have demonstrated that treatment with Ro5-4864, an inhibitor of mitochondrial membrane permeabilization, decreases intracranial pressure and consequently improves cerebral perfusion in experimental brain injury. This neuroprotective effect has been correlated with normalization of the extracellular markers of metabolic crisis (41). On the other hand, clinical studies have demonstrated that tight glycemic control and intensive insulin therapy result in increased cerebral metabolic crises in patients with head injury (42, 43). Thus, we may assume that an adapted energetic supply may limit metabolic crisis. This is the strategy chosen by Oddo in his ongoing clinical trials: “Lactate therapy after traumatic brain injury” (ClinicalTrials.gov Identifier: NCT01573507).

Protocol-driven management is currently used to treat most head injuries (44). This approach, which relies on evidence-based recommendations and particularly those collected from clinical trials, has led to improvements in the outcomes of head-injured patients (45). Nevertheless, one main concern regarding these clinical trials is that they are typically conducted on mixed categories of head-injured patients, regardless of their pathological state. As the underlying mechanisms for each type of post-traumatic metabolic disturbance are different, standard therapeutic interventions may not be equally efficient on patients that are (1) stabilized, (2) hyperglycolytic, (3) ischemic, or (4) experiencing metabolic crisis. Thus, there is a real need for clinical trials that would be conducted on homogenous populations of patients with a specific type of post-traumatic metabolic disturbance. As far as we known, no such specific clinical trial has ever been conducted, and especially not in patients with metabolic crisis. In addition to specific clinical trials, there would be clear benefits to shift to an approach targeting the individual needs of the patients (44). Indeed, individualized management may allow the adaptation of the treatment based on the metabolic state of the patient, at any time-point of the pathology. This kind of therapeutic strategy might be particularly helpful in the context of metabolic crisis.

The most important factor to consider in future clinical trials is the segregation of patients with metabolic crisis. To date, in everyday clinical practice, only multimodal monitoring, i.e., microdialysis and brain tissue oxygen (P_ti_O_2_) monitoring, has allowed the detection of metabolic crisis. However, these techniques do not meet the requirements for determination of a gold standard: sensitivity and specificity. Indeed, (1) the microdialysis markers of metabolic crisis are not sufficiently specific, and (2) P_ti_O_2_ primarily reflects a compromise between local CBF and oxygen delivery and therefore gives only very indirect information on cerebral oxygen metabolism (46). Moreover, multimodal monitoring generates a lot of data that requires specialized powerful software and adequate training in interpretation (17). So, because its implementation is expensive and time-consuming, not all ICUs are ready to use multimodal monitoring (47). Future research should establish a new gold standard for metabolic crisis that would be easy to deploy in all ICUs.

## Conclusion

Although metabolic crises are frequent and deleterious after traumatic brain injury, they have not been much studied so far. The challenge of the coming years will be to clearly define the specific mechanisms underlying metabolic crisis in order to improve its diagnosis and to optimize therapeutic treatment. Medical education about metabolic crisis will be a key factor for optimal management of head-injured patients with metabolic crisis.

## Conflict of Interest Statement

The authors declare that the research was conducted in the absence of any commercial or financial relationships that could be construed as a potential conflict of interest.
